# Persistent Thrombotic Hemangioma With Organizing/Anastomosing Features: A Case Report of a Guanine Nucleotide-Binding Protein Alpha Subunit (GNA)-Mutated Cutaneous Vascular Lesion

**DOI:** 10.7759/cureus.68446

**Published:** 2024-09-02

**Authors:** Svetlana Bobkova, Eli P Oldham, Patti Loykasek, Clifford L Henderson, Igor Shendrik

**Affiliations:** 1 School of Biomedical Sciences, Oklahoma State University Center for Health Sciences, Tulsa, USA; 2 Osteopathic Medicine, Oklahoma State University Center for Health Sciences, Tulsa, USA; 3 Molecular, Immunohistochemistry and Flow Cytometry, Pathology Laboratory Associates, Tulsa, USA; 4 Skin Cancer Surgery, Saints Dermatology Center of Excellence, Oklahoma City, USA; 5 Dermatopathology Section, Regional Medical Laboratory and Pathology Laboratory Associates, Tulsa, USA

**Keywords:** hemangioendothelioma, angiosarcoma, anastomosing hemangioma, next generation sequencing (ngs), thrombotic hemangioma

## Abstract

Thrombotic hemangioma with organizing/anastomosing features (THOA) is a newly identified variant within the spectrum of hemangiomas that harbor mutations in the guanine nucleotide-binding protein alpha subunit (GNA) genes (like *GNAQ* or *GNA11*). While THOA shares similarities with anastomosing hemangioma, it possesses distinct clinical and morphological characteristics that make it a separate entity. All reported cases of THOA have demonstrated benign behavior. However, histologic features such as anastomosing vascular growth, mitotic figures, and endothelial hobnailing may raise concerns for a low-grade malignant vascular neoplasm.

We report the case of a 74-year-old female with an unremarkable medical history who presented with a vascular lesion on her upper torso. The lesion persisted after the initial biopsy and was re-excised, displaying similar histologic characteristics. Next-generation sequencing (NGS) revealed a *GNAQ* mutation (p.Q209H) in both samples. Notably, a *TP53* mutation (p.R273H) was detected in the first specimen but was absent in the subsequent excision. The lesion was diagnosed as persistent THOA. This case report discusses the salient features, genetic profile, and prognosis of this uncommon lesion.

## Introduction

Thrombotic hemangioma with organizing/anastomosing features (THOA) has been recently introduced as a provisional entity closely related to anastomosing hemangioma (AH) [1,2]. Both lesions are benign and share histologic features, such as anastomosing growth of sinusoidal capillaries, fibrin thrombi, and hobnailed endothelial cells; however, THOA exhibits a strong predilection for the dermis and subcutis of the lower abdominal region [1,2]. In contrast, most AH cases have been reported in visceral sites, including genitourinary organs, liver, and gastrointestinal tract [2]. To summarize, THOA and AH represent the same lesion occurring in different anatomic locations.

Both neoplasms have been shown to harbor guanine nucleotide-binding protein alpha subunit (GNA) mutations, though the current data is insufficient to determine the frequency of specific mutations within these lesions due to the limited number of reported cases [1,2]. Therefore, our report aims to contribute to the growing body of literature regarding the genetic profile of THOA with the use of next-generation sequencing (NGS).

## Case presentation

A 74-year-old woman with no significant medical history-specifically, no history of breast pathology or radiation therapy-presented with a vascular lesion located on the right side of her torso, near the right breast. The lesion exhibited a raised, dark blue superficial component and a firm, deep dermal component, measuring approximately 18 mm in total. The lesion was well-demarcated, with a smooth, slightly elevated contour and no visible signs of ulceration or bleeding.

**Figure 1:**
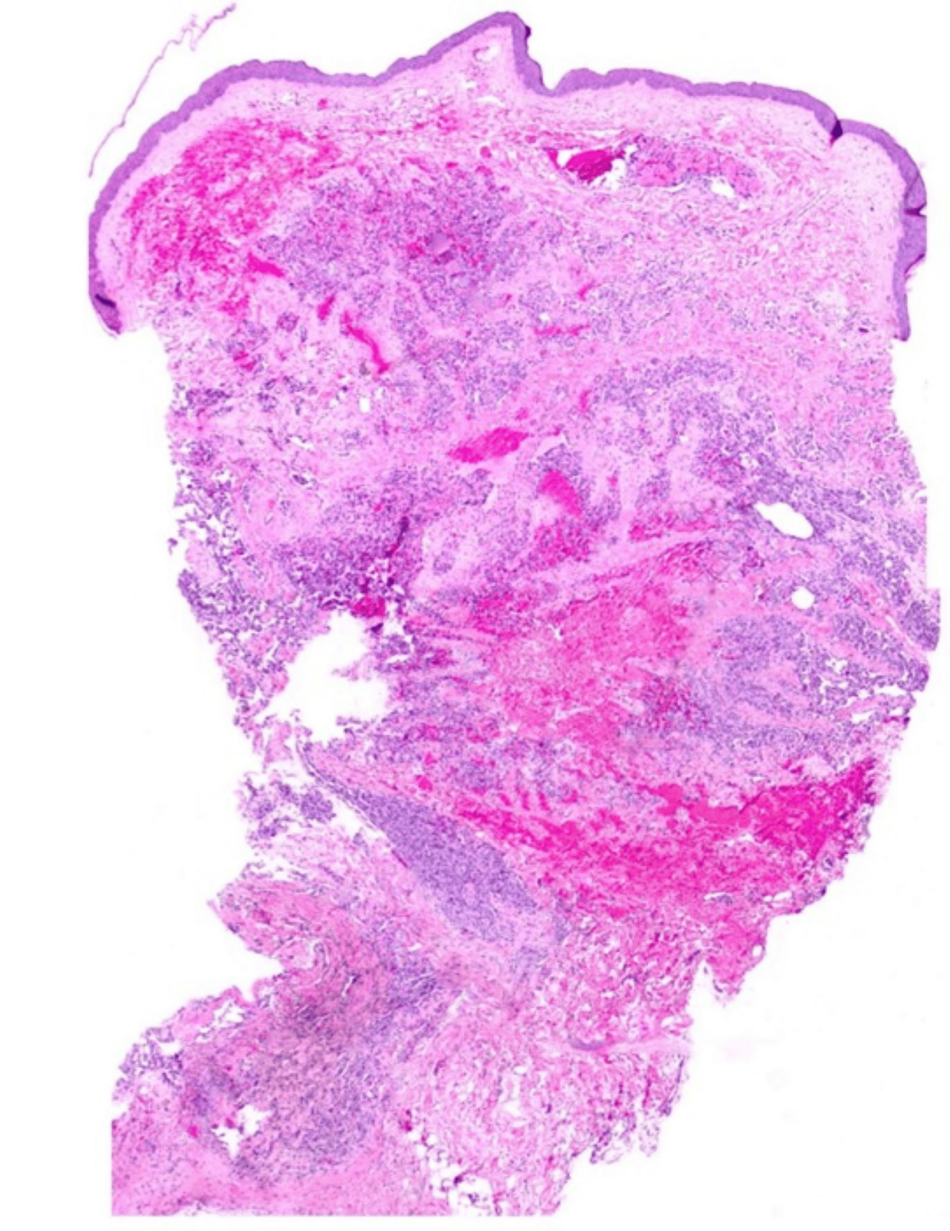
Punch biopsy of skin involved by lobulated vascular neoplasm with areas of thrombosis, hemorrhage, and hemosiderin deposition (20x magnification).

**Figure 2:**
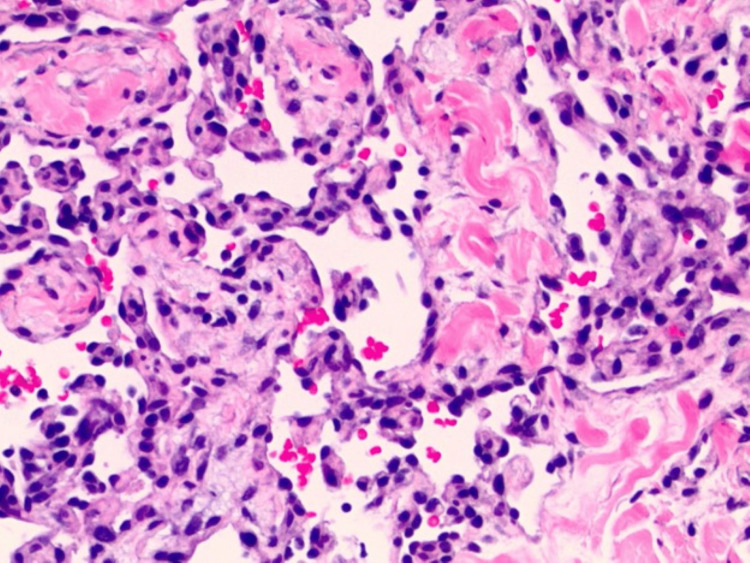
Anastomosing vascular channels with endothelial cells exhibiting hobnail morphology, papillary-like structures, and hyalinization of the vascular walls (200x magnification).

**Figure 3:**
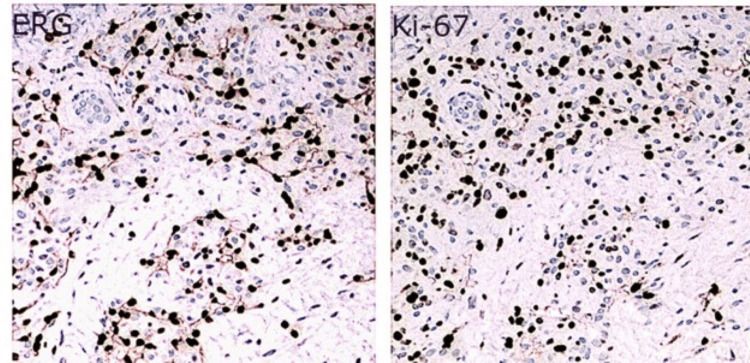
Parallel images of ERG (left) and Ki-67 (right) immunohistochemical stains demonstrating numerous Ki-67 positive cells that do not colocalize with ERG-positive endothelial cells (100x magnification). ERG: ETS-related gene.

A punch biopsy of the lesion revealed a vascular growth with a variegated appearance, predominantly composed of lobular proliferation of capillary-sized vessels. The upper portion of the lesion contained areas of thrombosis and scattered foci of red blood cell extravasation. Additionally, there was an ill-defined area of hemosiderin deposition observed in the deeper dermis (Figure 1). High-power examination showed collections of anastomosing vascular spaces lined by prominent, hyperchromatic endothelial cells, many of which exhibited hobnail morphology (Figure 2). The vascular walls were partially hyalinized, with no extramedullary hematopoiesis identified. Rare mast cells were observed, approximately 3 per mm^2^, although specific evaluation with CD117 staining was not performed. Immunohistochemical staining with ETS-related gene (ERG) and Ki-67 confirmed the vascular nature of the neoplasm and demonstrated brisk Ki-67 positivity, although not all Ki-67-positive cells colocalized with ERG-positive endothelial cells (Figure 3). Additionally, immunohistochemical staining for HHV8 was performed and yielded negative results. The initial diagnosis was atypical vascular proliferation.

**Figure 4:**
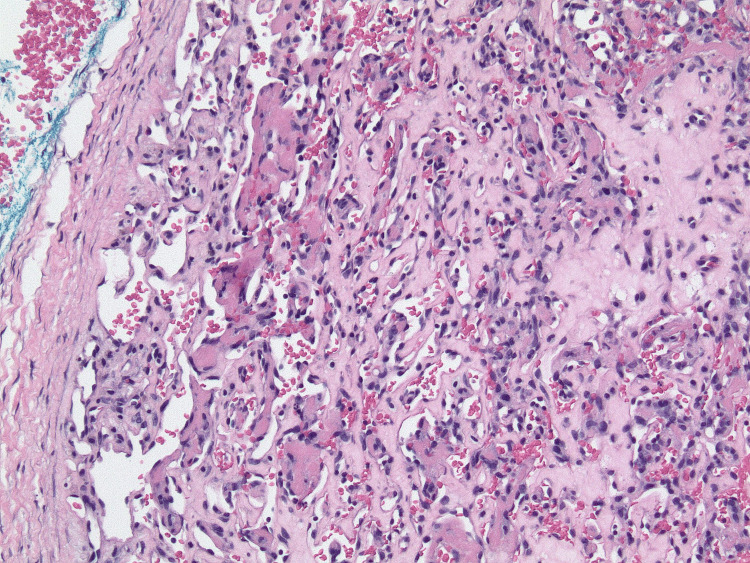
Microthrombi in the deep portion of the lesion with adjacent areas of hyalinization (100x magnification).

The lesion remained clinically persistent, leading to a second excisional biopsy fifteen months later. This biopsy revealed similar lesional morphology with anastomosing vascular spaces, endothelial cell hobnailing, areas of thrombosis, and more prominent fibrosis with endothelial cell entrapment within the hyalinized stroma. The biopsy revealed areas with up to six mitoses per high-power field (HPF), indicating increased proliferative activity. The presence of deep lesional thrombosis appeared unrelated to mechanical trauma and likely represents a salient feature of this neoplasm (Figure 4). Next-generation sequencing (NGS) was performed on both specimens, revealing a *GNAQ *mutation (p.Q209H) in both samples. The variant allele frequency (VAF) of the *GNAQ *mutation was present at 9.3% in the first sample and increased to 18.3% in the second sample. Additionally, a *TP53 *mutation (p.R273H) was detected in the first sample with a VAF of 3.2%, but this mutation was absent in the second.

The patient experienced an uneventful recovery following the second biopsy of the lesion, with no complications observed during the subsequent follow-up period.

## Discussion

Most adult cutaneous vascular neoplasms are benign hemangiomas, primarily composed of endothelial cells. Between benign hemangiomas and high-grade angiosarcomas lies a category of neoplasms characterized as low-grade angiosarcomas, both histologically and likely biologically. The term "hemangioendothelioma" was introduced to describe these intermediate-grade neoplasms [3]. Differentiating certain hemangioma variants from hemangioendotheliomas and low-grade angiosarcomas can be challenging due to overlapping histologic features.

A key histologic feature of aggressive endothelial neoplasms is the presence of anastomosing vascular channels, especially when accompanied by any degree of endothelial cell atypia. Although such features can appear in some benign lesions, these are often classified as distinct entities, such as Masson's tumor, to differentiate them from their malignant counterparts.

One such recently defined entity is AH, first described in 2009 by Montgomery and Epstein [2]. AH is a rare, histopathologically distinct, benign vascular neoplasm that mimics well-differentiated angiosarcoma and predominantly affects the genitourinary tract and paraspinal region [2]. The tumor typically presents as a well-delineated but unencapsulated mass with a spongy gross appearance. Histologically, AH is characterized by anastomosing growth of sinusoid capillaries, resembling the splenic red pulp. Other common features include fibrin thrombi, eosinophilic hyaline globules, extramedullary hematopoiesis, and hobnailed endothelial cells. The presence of anastomosing growth patterns and possible nuclear atypia can lead to confusion with angiosarcoma. However, AH consistently demonstrates benign clinical behavior, with no reported cases of metastasis.

AH has been predominantly reported in visceral sites, and no case of cutaneous AH has been documented in the literature [4]. Additionally, a series of closely related neoplasms, termed THOAs, has been recently described, expanding the spectrum of vascular lesions with anastomosing features [1]. Due to atypical histologic features such as hobnailing, anastomosing vascular channels, and rare mitoses, these lesions may have previously been diagnosed descriptively as vascular neoplasms with atypical features. The rarity of published cases of cutaneous AH and THOA contributes to diagnostic challenges and limited awareness of this benign hemangioma variant.

Advances in molecular techniques, particularly the increased availability of next-generation sequencing (NGS), have significantly expanded the spectrum of vascular tumors. This has been especially impactful in identifying novel gene fusions at the benign and low-grade end of the spectrum, enhancing classification and understanding of these entities [5].

The genetic hallmark of AH is *GNAQ* hotspot mutations at codon 209 in 45-69% of cases, while other less common genotypes have been reported, such as* GNA14* and *GNA11* mutations [5].

*GNAQ*, *GNA14*, and *GNA11* encode guanine nucleotide-binding protein (G-protein) subunits that are part of the alpha-q subfamily of G-proteins, with *GNA14* and* GNA11* being paralogs of *GNAQ* [6]. These G-proteins mediate the signaling of G-protein coupled receptors (GPCRs) by coupling the seven transmembrane domain receptors on the cell membrane to intracellular signaling pathways, including the phosphoinositide 3-kinase (PI3K)/AKT and mitogen activated protein kinase (MAPK) pathways [6]. This signaling cascade results in the activation of MAP kinase pathways, which control cell proliferation, survival, and protein synthesis [7].

The alpha subunit of heterotrimeric G-proteins, such as *GNAQ*, functions as a molecular switch [8]. It is active when bound to guanosine triphosphate (GTP) and inactive when GTP is hydrolyzed to guanosine diphosphate (GDP) [8]. Substitutions of specific glutamine or arginine residues in the alpha subunit that interact with the GTP molecule can block its intrinsic GTPase activity, thereby locking the G-protein in a constitutively active GTP-bound state [8,9].

Activating mutations in codon 209 of *GNAQ* and related genes result in this constitutive activation and are implicated as drivers in tumorigenesis [10]. These mutations are significant in human neoplasia, being present in uveal and meningeal melanocytic tumors, blue nevi, vascular malformations including Sturge-Weber syndrome, and several types of hemangiomas, such as congenital hemangioma, anastomosing hemangioma, and hepatic small vessel neoplasm [11].

In this case report, we describe two sequential biopsies of a persistent cutaneous hemangioma with an unusual combination of anastomosing vascular channels, endothelial hobnailing, lobular growth pattern, and large areas of thrombosis, which appear unrelated to mechanical trauma. The lesion has a variegated appearance, with areas of organizing thrombosis and hyalinization adjacent to regions without thrombi that display a more prominent anastomosing vascular pattern. The histologic appearance of both specimens is similar, suggesting that thrombosis is an intrinsic feature of the lesion rather than a result of mechanical trauma.

Both specimens demonstrated a *GNAQ *mutation with the amino acid (AA) change p.Q209H, where glutamine (Q) is substituted by histidine (H) at position 209. This is a missense mutation. Recent studies have reported the presence of mutually exclusive mutations in* GNA14 Q205*,* GNA11*, and* GNAQ Q209* in benign hemangiomas, but not in malignant vascular tumors [7], which supports the histologic impression of THOA. Notably, a low-frequency TP53 mutation (AA change p.R273H) was detected in the first sample but absent in the second. While TP53 mutations are observed in some aggressive vascular neoplasms, this finding appears clinically insignificant, in this case, due to its low frequency and the mutation's absence in the subsequent sample.

The histologic appearance of THOA differs from low-grade angiosarcoma or hemangioendothelioma by the presence of thrombosis, lobular growth pattern, and predominantly pushing rather than infiltrative expansion [1]. Although the differential diagnosis includes intravascular papillary endothelial hyperplasia (Masson's tumor), the latter is typically characterized by zonation, circumscription, and frequent evidence of intravascular localization, even in small tissue samples. Traumatized cherry hemangiomas may also exhibit somewhat similar histologic features, particularly in superficial biopsies, necessitating clinical-pathologic correlation and a larger sample that includes deeper dermal layers.

## Conclusions

This report describes a case of thrombotic hemangioma with organizing/anastomosing features (THOA), a lesion closely related to the histologically similar anastomosing hemangioma found in internal organs. Both lesions are newly described entities with a limited number of published cases. In addition to thrombosis, THOA is characterized by anastomosing vascular channels and endothelial hobnailing, which may raise concerns about an aggressive neoplasm. However, the benign nature of the lesion was supported by the identification of the* GNAQ* Q209 mutation. Increased awareness of this unusual vascular neoplasm is crucial to avoid overtreatment.
